# Supplementary material for: On the impact of capillarity for strength at the nanoscale
Source: Nat Commun. 2017 Dec 7;8:1976. doi: 10.1038/s41467-017-01434-2 (PMC5719409; doi:10.1038/s41467-017-01434-2)
Supplement: Supplementary file 1 — Supplementary Information [file 41467_2017_1434_MOESM1_ESM.pdf]

Supplementary Note 1: Origin of equations for surface-induced stress

Equations (4) in the main text follow from Equation (20) of reference [1] when firstly, setting – in line with the present assumption of isotropic surface stress – the axial and radial surface stress components as equal and secondly, correcting the obvious (surface and bulk stresses need to cancel, which is the central theme in reference [1]) omission of a minus sign in front of the stress.

Supplementary Note 2: Magnitude of reduction in surface tension due to relaxation

The magnitude of the reduction in surface tension, γ, due to relaxation may be estimated as follows: Take the example of a gold nanowire with 111-type surfaces, where roughly $\gamma = 1.1$ N/m, $f = 3.3$ N/m [2], $Y = 80$ GPa and $\nu = 0.42$ [3]. Even for a nanowire with r as small as 1 nm, the second term in the bracket of Equation (7) is only 0.12 N/m, leaving the first term, γ, as the dominant contribution. Samples in the experimental part of our work have $r \sim 20$ nm, so that the impact of relaxation on γ may be safely neglected.

Supplementary Note 3: Work of deformation

When a dislocation line segment d**l** is displaced in its glide-plane by the vector $\delta\mathbf{s}$, then the work done against the local acting stress $\mathbf{S}^{\rm net}$ is $\delta W = (\mathbf{S}^{\rm net}\cdot\mathbf{n}\delta A)\cdot\mathbf{b}$, where **b** denotes the Burgers vector and $\mathbf{n}\delta A = \mathrm{d}\mathbf{l} \times \delta\mathbf{s}$ with δA the area covered [4]. Therefore, when a glide-plane of finite area is swept by the dislocation, the net work scales with the area-integral of $\mathbf{S}\cdot\mathbf{n}$, the traction.

Supplementary Note 4: Cyclic voltammetry of nanoporous gold in different electrolytes

Supplementary Figure 1 shows cyclic voltammograms of nanoporous gold (NPG) in the electrolytes of our study. This data was used for defining the interval of nominally capacitive charging, for use in the in situ compression tests.

Supplementary Note 5: In situ compression tests during capacitive processes

On top of the in situ compression tests shown in the main text, additional tests on NPG samples were performed in smaller potential intervals, excluding the region of oxygen electrosorption. The purpose of these experiments was to verify that *i)*, the sign inversion of the flow-stress potential response is recovered when the potential step sequence is inverted and that *ii)*, consistent results for the response are also obtained in the initial stages of plastic deformation, at small strain. Supplementary Figure 2 illustrates the results of an experiment with mean ligament size $L = 40$ nm in 1 M $HClO_4$ and of another experiment with $L = 30$ nm in 0.5 M H_2SO_4. Parts **a** and **b** of the figure show the stress-strain curves (red graphs) along with the potential step protocols (blue graphs). In both compression tests the potential scan started with a hold at the potential of zero charge, $E_{\rm zc}$, for NPG in the respective electrolyte. The potential, E, was then decreased to a more negative value and subsequently stepped upward (anodic) to an upper vertex of 1.0 V. In Supplementary Fig. 2a the scan direction was then inverted, so that the potential step series continued negative-going (cathodic). In Supplementary Fig. 2b the potential cycles were started at lesser strain, at about 4% of engineering strain.

As can be seen from Supplementary Fig. 2c, the trends for the flow-stress potential coupling parameter $\delta\sigma/\delta E$ versus E are indeed reproducible on the anodic and cathodic step series: Consistent with the graph for 1 M $HClO_4$ in Fig 6a of the main text, the coupling approximates zero when $E_{\rm zc}$ is approached from below, and the behavior is consistent during the backwards scan. The numerical values of the coupling during anodic and cathodic scan differ. This is consistent with the flow stress increase during ongoing compression and with our observation, see the main text, that the coupling scales with the net value of the flow stress. In fact, the two branches coincide in the plot of the normalized response parameter, $\sigma^{-1}\delta\sigma/\delta E$, in Supplementary Fig. 2d.

Supplementary Figure 2b shows that the findings from the main text apply also when the potential jumps

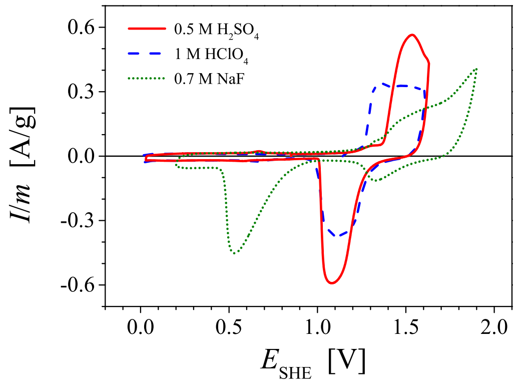

Supplementary Fig. 1: Cyclic voltammograms for mean ligament diameter of 40 nm. Experiments with nanoporous gold in 0.5M H_2SO_4, 1M $HClO_4$, and 0.7M NaF. Scan rate 5 mV/s. Current I is normalized to a sample mass m in each single experiment.

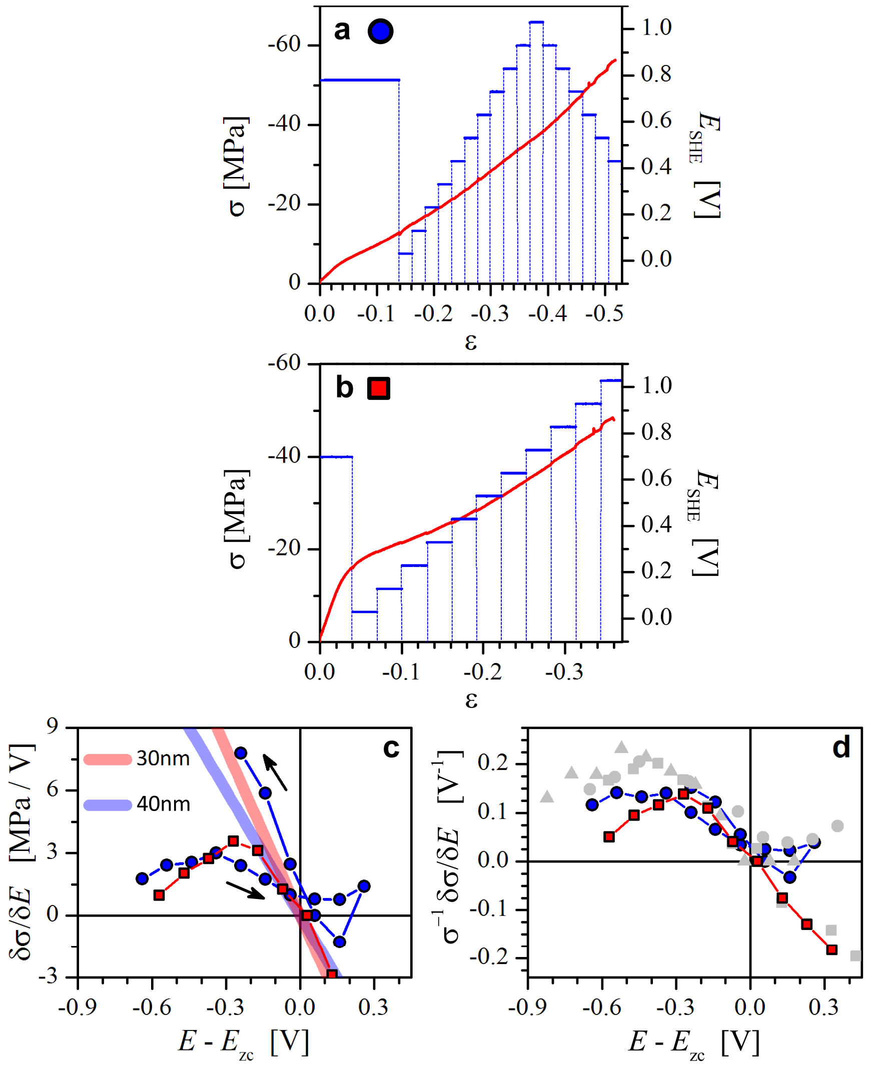

Supplementary Fig. 2: In situ compression tests with different potential step protocols. Nanoporous gold at constant engineering strain rate of 10^{-5} s^{-1}. **a** Test for a sample with ligament size $L = 40$ nm in 1 M $HClO_4$, with potential steps exploring anodic (positive-going) and reversed cathodic directions. Red: graph of stress σ versus strain ε; blue: electrode potential E versus the standard hydrogen electrode (SHE). **b** Test as in a), but for $L = 30$ nm and in 0.5 M H_2SO_4, here with potential jumps (in anodic direction) starting already at strain 4%. **c** Flow stress-potential response, $\delta\sigma/\delta E$, versus the potential, $E - E_{zc}$, relative to the potential of zero charge, from the experiments in a) (blue circles) and in b) (red squares). Arrows show directions of the potential steps. Bold lines: predicted coupling strength near E_{zc} from Equation (12) of the main text, using the capacitance value $c = 40$ $\mu F/cm^2$ and ligament sizes as indicated in legend. **d** Normalized flow stress response versus potential. Grey symbols: data from the in situ tests compiled in Fig. 6 of the main text. Note the mutual consistency of all data sets, irrespective of ligament size and electrolyte.

are started at an earlier stage of deformation, namely already at 4% strain. Again, the flow-stress potential response – here in 0.5 M H_2SO_4 – is negative for $E < E_{zc}$ and positive for $E > E_{zc}$.

A comparison of the absolute values of the normalized response parameter with those of Fig 6a in the main text (grey symbols in Supplementary Fig. 2d) shows excellent agreement.

As in the main text, we also compared the experimental coupling parameters to the theory of Equation (12), which is represented by the solid lines in Supplementary Fig. 2c. For the experiment of Supplementary Fig. 2b the response near the pzc is well compatible with the theory. The data derived from the experiment of Supplementary Fig. 2a shows a smaller-than-predicted $\delta\sigma/\delta E$ during the anodic potential scans, whereas the

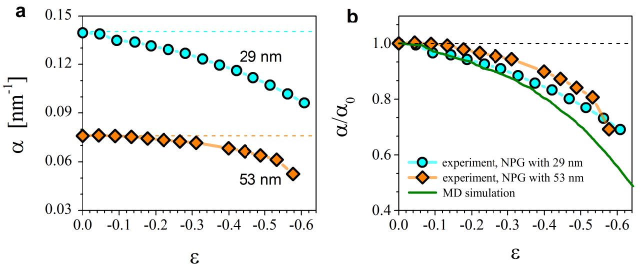

Supplementary Fig. 3: Surface area variation of nanoporous gold during compression. **a** Evolution of volume-specific surface area (per solid volume), α, with compressive strain ε for two different ligament sizes as measured by electrochemical impedance spectroscopy technique in 1 M $HClO_4$. **b** Comparison with changes in α of "dry" NPG upon compression as obtained by molecular dynamics simulations (MD) in Supplementary ref. [8] (green solid line). Both experimental and MD values have been scaled to the starting value of α before compression, α_0. Dashed lines emphasize initial value, solid lines are guides to the eye. Note the excellent agreement between experiment and MD results.

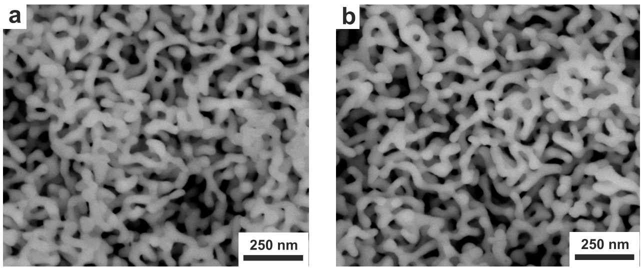

Supplementary Fig. 4: Evolution of microstructure during potential jump series. Scanning electron micrographs of microstructure before (a) and after (b) applying potential jumps representative of a mechanical in-situ tests. Potential-time protocol in 1 M $HClO_4$ was identical with Supplementary Fig. 2 and with Fig. 4 of the main text, except that the series here ended after 1.0 V was reached. This replicates the variation in the capacitive regime, which underlies Fig. 6a in the main text and the discussion of capacitive processes there. Parts **a** and **b** image two opposing faces of a cleavage surface of the same sample, treated differently. Analysis of the mean ligament size in the two images supplied the values 41.7 and 43.1 nm, respectively.

later, cathodic series gives a stronger response than predicted. In view of the good agreement of the normalized parameters with those in Fig 6a we conclude that the behavior of these two samples is consistent with the reports in the main text, except for a somewhat lesser or greater flow stress.

Supplementary Note 6: Surface area variation during compression

Supplementary Figure 3a displays the surface area, A, that was evaluated in situ during compression in 1 M $HClO_4$. The experimental procedure was as follows: We used the capacitance ratio method [5], which determines A from $A = C/c_{DL}$ with C the net capacity and c_{DL} the specific double-layer capacitance, $c_{DL} = 40\ \mu F/cm^2$ [6]. Electrochemical impedance spectrometry (EIS) in the frequency range $0.1 - 1$ Hz and at the DC potential 0.8 V provided C via the procedures from Ref [7]. The impedance spectra were run continuously during the whole deformation experiment and the DC potential of 0.8 V was shortly $(60 - 80$ s) imposed on the sample during recording of each single EIS data point. The values of A were then normalized by the solid volume (sample mass divided by mass density of gold) of the NPG samples.

The graphs of A versus the compressive strain in Supplementary Fig. 3a show a consistent reduction of area during plastic deformation, consistent with a key assumption in our theory. The graph of relative change in surface area during compression, Supplementary Fig. 3b, affords a verification of the consistency. Superimposed to the data is here a graph representing the molecular dynamics simulation (MD) of plastic deformation of NPG during compression in Supplementary ref. [8] (Fig. 5b there). It is seen that the graphs from the experiment and simulation are in good agreement, specifically in the early stages of compression. Note that the area may be affected by the formation of new contacts between ligaments through cold welding. Yet, this process is expected to prevail only at the later stages of compression. The variation in surface area is therefore significant as an indication of thickening of the ligaments during compression, see our theory.

It is known that exposure to electrolyte and potential can leads to coarsening of the ligaments of NPG [9, 10]. Changes in microstructure before and after applying the potential steps of the experiment in Supplementary Fig. 2 have been explored in the following way: An as-prepared sample was cleaved and one of the two halves was exposed to potential cycles as in an in situ compression test, whereas the other saw no contact with electrolyte. Both halves were then investigated side-by-side in a scanning electron microscope and the identical image analysis was used to quantify a distribution of sizes. Supplementary Figures 4a and b shows the corresponding scanning electron micrographs (SEMs). No change in ligament diameter is apparent to the naked eye.

For a precise assessment of possible coarsening, we performed a stereologic analysis of the images by the image processing software ImageJ [11]. SEMs of larger areas, containing roughly 1000 ligaments, were binarized using identical threshold values and the resulting images were then analyzed by ImageJ's BoneJ Thickness plugin [12, 13, 14]. In its implementation for 2D

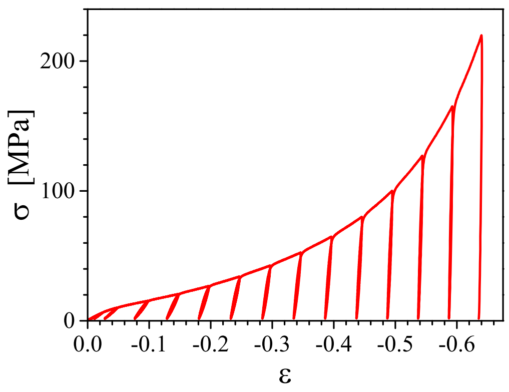

Supplementary Fig. 5: Load-unload stress-strain data. Compression tests of dry NPG with ligament size $L = 40$ nm. Engineering strain rate 10^{-4} s^{-1}. Note that yielding at the beginning of reload segment coincides with the last flow stress value prior to the unload.

images, the algorithm uses the method of Supplementary ref. [15], computing a local size measure as the diameter of the largest inscribed circle at any point and specifying the mean size as the average of the diameter distribution.

Analysis of the mean ligament size in the images of Fig 4a and b supplied the values 41.7 and 43.1 nm, respectively. In view of the microstructural heterogeneity that is present even in the quite well-defined structure of NPG, the two numbers may be considered as consistent with little, if any, coarsening. The relative change in ligament size is certainly smaller than the relative change in specific surface area of Supplementary Fig. 3. This implies minimal or no effect of the applied potentials on the porous network in our study. The observed decreasing of the surface area may thus be dominantly attributed to the compressive strain.

Supplementary Note 7: Link between flow stress and yield strength in nanoporous gold

A connection between flow stress and yield strength of NPG is very well illustrated by a compressive stress-strain diagram with unload-reload cycles (Supplementary Fig. 5). In the compression test, a dry sample with ligament diameter of about 40 nm was continuously loaded with engineering strain rate of 10^{-4} s^{-1} and subjected to intermediate load/unload segments. During reloading, a strain level was incrementally increased followed by unloading up to a minimum stress value of ~ 2 MPa. The further details of the experiment can be found in Supplementary ref. [16]. Upon reloading the NPG specimen suffered plastic yielding at the flow stress value that was reached just before the unload. This implies that the flow stress at a certain state of plastic strain represents the yield strength of the material in that strain state. Thereby, it emphasis a strong coupling between strength and plastic flow in NPG.

Supplementary References

[1] J. Weissmüller and J. W. Cahn. Mean stresses in microstructures due to interface stresses: A generalization of a capillary equation for solids. *Acta Materialia*, 45(5):1899–1906, 1997.

[2] Y. Umeno, C. Elsässer, B. Meyer, P. Gumbsch, M. Nothacker, J. Weissmüller, and F. Evers. Ab initio study of surface stress response to charging. *Europhysics Letters*, 78(1):13001, 2007.

[3] W.F. Gale and T.C. Totemeier, editors. *Smithells Metals Reference Book*. Elsevier Butterworth-Heinemann, 8th edition, 2004.

[4] P.M. Anderson, J.P. Hirth, and J. Lothe. *"Theory of Dislocations"*, chapter 4.6 and 20.5. Cambridge University Press, New York, 3rd edition, 2017.

[5] S. Trasatti and O.A. Petrii. Real surface area measurements in electrochemistry. *Journal of Electroanalytical Chemistry*, 327(1-2):353–376, 1992.

[6] P.S. Germain, W.G. Pell, and B.E. Conway. Evaluation and origins of the difference between double-layer capacitance behaviour at Au-metal and oxidized Au surfaces. *Electrochimica Acta*, 49(11):1775–1788, 2004.

[7] E. Rouya, S. Cattarin, M. L. Reed, R. G. Kelly, and G. Zangari. Electrochemical characterization of the surface area of nanoporous gold films. *Journal of The Electrochemical Society*, 159(4):K97–K102, 2012.

[8] B.-N. D. Ngô, A. Stukowski, N. Mameka, J. Markmann, K. Albe, and J. Weissmüller. Anomalous compliance and early yielding of nanoporous gold. *Acta Materialia*, 93:144–155, 2015.

[9] Y. Ding, Y.-J. Kim, and J. Erlebacher. Nanoporous gold leaf: Ancient technology Advanced material. *Advanced Materials*, 16(21):1897–1900, 2004.

[10] A. Sharma, J.K. Bhattarai, A.J. Alla, A.V. Demchenko, and K.J. Stine. Electrochemical annealing of nanoporous gold by application of cyclic potential sweeps. *Nanotechnology*, 26(8):085602, 2015.

[11] C.A. Schneider, W.S. Rasband, and K.W. Eliceiri. NIH Image to ImageJ: 25 years of image analysis. *Nature Methods*, 9(7):671–675, 2012.

[12] M. Doube, M.M. Klosowski, I. Arganda-Carreras, F.P. Cordeliéres, R.P. Dougherty, J.S. Jackson, B. Schmid, J.R. Hutchinson, and S.J. Shefelbine. BoneJ: Free and extensible bone image analysis in ImageJ. *Bone*, 47(6):1076–1079, 2010.

[13] R. Dougherty and K. H. Kunzelmann. Comput-

ing local thickness of 3D structures with ImageJ. *Microscopy and Microanalysis*, 13(S02):1678–1679, 2007.

[14] T. Hildebrand and P. Rüegsegger. A new method for the model-independent assessment of thickness in three-dimensional images. *Journal of Microscopy*, 185(1):67–75, 1997.

[15] N.J. Garrahan, R.W.E. Mellish, S. Vedi, and J.E. Compston. Measurement of mean trabecular plate thickness by a new computerized method. *Bone*, 8(4):227–230, 1987.

[16] N. Huber, R. N. Viswanath, N. Mameka, J. Markmann, and J. Weißmüller. Scaling laws of nanoporous metals under uniaxial compression. *Acta Materialia*, 67(0):252–265, 2014.
